# Differences between manufacturer-specified and measured effective inner diameters of vascular introducer sheaths: a micro-CT analysis

**DOI:** 10.1186/s42155-026-00714-7

**Published:** 2026-06-06

**Authors:** Franz Wegner, Vanessa Vaino, David Melenberg, Malte M. Sieren, Thomas Friedrich, Thorsten M. Buzug, Maik Stille, Roman Kloeckner

**Affiliations:** 1 Institute of Interventional Radiology, University of Lübeck, Lübeck, Germany; 2 Fraunhofer Research Institution for Individualized Medical Technology and Engineering IMTE, Lübeck, Germany; 3 TH Lübeck, Lübeck, Germany; 4 Institute of Radiology and Nuclear Medicine, University of Lübeck, Lübeck, Germany; 5 Institute of Medical Engineering, University of Lübeck, Lübeck, Germany

**Keywords:** Endovascular devices, Introducer sheaths, Nondestructive testing, Micro-CT

## Abstract

**Purpose:**

To quantify deviations between the inner diameters specified by manufacturers and the effective diameter of vascular introducer sheaths.

**Materials and methods:**

A total of 18 different introducer sheaths (one device per type) with nominal inner diameters (nID) of 5 French (Fr), 6 Fr, and 7 Fr and lengths of 10 cm, 25 cm, and 45 cm, manufactured by four different vendors (Terumo, Biotronik, Merit, and Cook Medical) were evaluated. Non-destructive testing was performed using a commercial Micro-CT system (Comet Yxlon) at three distinct anatomical locations: the hub, middle, and tip/aperture. Cylindrical and circular least-squares fits were applied to calculate the effective inner diameters (eID). The eIDs were compared with the inner diameters specified by the manufacturer (mID) and the nominal inner diameter (nID) in Fr, which both are regularly mentioned for each product.

**Results:**

All non-braided sheaths revealed eIDs at the hubs, middles, and tips exceeding the mIDs (up to + 8.5%). All eIDs at the sheath aperture were consistently smaller than the mIDs and, with one exception, smaller than the nID. For braided sheaths, most eIDs at the hub were up to 3.4% larger than specified. However, at the tips and the apertures all braided sheaths were smaller than the mID (up to − 10.99%). In comparison with the nID, most braided sheaths were larger.

**Conclusion:**

Relevant deviations exist between the effective and manufacturer-specified inner diameters of vascular sheaths, especially at the tips. These discrepancies may necessitate the selection of larger sheath sizes to accommodate specific interventional devices, particularly when aperture constraints are critical.

**Supplementary Information:**

The online version contains supplementary material available at 10.1186/s42155-026-00714-7.

## Introduction

Vascular introducer sheaths are among the most essential tools for endovascular procedures. Sheaths provide minimal-invasive and constant vascular access for the insertion of various endovascular devices. The diameter of the introduced sheath normally is chosen as small as possible considering the anticipated device portfolio and the given patient anatomy [1]. The length of the chosen sheath is mainly influenced by the distance to the vascular target region. Non-braided sheaths are used for short and straight vascular access sites, whereas braided sheaths, with a metallic support material that prevents sheath buckling, are applied for curvy vasculature, e.g., cross-over maneuvers.

The nominal sheath size, which is given by the manufacturer, reflects the available inner diameter (ID), specified in French (1 Fr, = 1/3 mm). In clinical routine, unexpected resistance during catheter insertion/removal or impaired device steerability may occur despite nominal size compatibility, particularly when combining devices from different manufacturers. Especially, when inserting stent delivery-systems or removing balloon catheters after angioplasty, the available sheath diameter is essential for a low-friction removal of the device. Regarding the choice of sheath sizes, recommendations given by the manufacturers are very broad and partially unspecific. The spectrum of recommendations in the IFUs reaches from “the introducer set chosen will be one or two French sizes larger than the French size of the balloon catheter” [2] to “choose a sheath size large enough to accommodate the maximum outer diameter of any devices that will be placed through the sheath” [3]. Notably, when considering an increasing complication risk with increasing sheath diameter [4], the approach of oversized sheaths is only limited justifiable from a medical point of view.

To our knowledge, manufacturer-reported inner diameters are derived from exemplary, standardized measurements and potentially not fully represent the effective lumen geometry at each position of a sheath. Local lumen reductions can result from, e.g., manufacturing tolerances, variations of material properties, and production inconsistencies. Macroscopic, mechanical measurement approaches are limited in their ability to detect small size variations and are potentially linked to deformation or even destruction of the device.

Micro-focus Industrial Computed Tomography (Micro-CT) enables high-resolution, non-destructive, three-dimensional characterization of medical devices. It has been successfully applied for the geometric analysis of catheters, stents, and other medical devices [5–7]. Taken together, Micro-CT offers ideal features to study the geometry of sheaths with very high spatial resolution (up to 4 µm). Whereas variations of the outer diameter of sheaths are reported [8], a systematic evaluation of the effective inner diameter is missing in the existing literature.

The purpose of this study was to quantitatively assess the effective inner diameter of commercially available vascular introducer sheaths using high-resolution Micro-CT and to compare these measurements with manufacturer-reported values.

## Material and methods

### Introducer sheaths

**Fig. 1:**
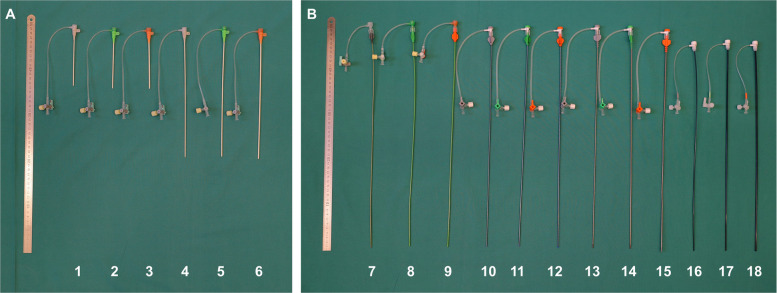
Photographs of all tested sheaths. In **A**, the non-braided sheaths with lengths of 10 cm and 25 cm are shown. In **B**, braided sheaths with a length of 45 cm are displayed

To study a representative device portfolio, 18 commercial vascular introducer sheaths in three commonly used diameter sizes (5 French (Fr), 6 Fr, and 7 Fr) were investigated. In this context, it must be noted that in addition to the nominal inner diameter (nID), the manufacturers provide a product-specific inner diameter (mID). All sheaths were manufactured by four different companies (Table 1, Fig. 1). The sheaths were all straight shaped and had lengths of 10 cm (*n* = 3), 25 cm (*n* = 3), and 45 cm (*n* = 12). The devices with lengths of 10 cm and 25 cm were completely made from non-metallic materials, whereas 45 cm sheaths were reinforced with metallic braiding between polymer layers. All sheaths were studied in a dry, controlled environment with a relative room humidity (RH) between 20 and 50% and a Micro-CT chamber temperature of 19–22 °C (maximum deviation within 1 h: 1 K and 3% RH, respectively), within a class 3 measuring room according to VDI/VDE 2627 [9].

**Table 1:** Overview of the sheath specifications including proprietary sheath name, inner diameter in mm given by the manufacturer (mID), nominal inner diameter (nID) in French (Fr), an introduced abbreviation, and information regarding the existence of sheath braiding

No.	Proprietary sheath name	mID [mm]	nID [Fr]	Sheath length [cm]	Abbreviation	Braiding
1	Terumo Radifocus Introducer II	1.780	5	10	5F10T	No
2	Terumo Radifocus Introducer II	2.100	6	10	6F10T	No
3	Terumo Radifocus Introducer II	2.450	7	10	7F10T	No
4	Terumo Radifocus Introducer II	1.780	5	25	5F25T	No
5	Terumo Radifocus Introducer II	2.100	6	25	6F25T	No
6	Terumo Radifocus Introducer II	2.450	7	25	7F25T	No
7	Terumo Destination	1.920	5	45	5F45T	Yes
8	Terumo Destination	2.210	6	45	6F45T	Yes
9	Terumo Destination	2.570	7	45	7F45T	Yes
10	Biotronik Fortress Introducer	1.950	5	45	5F45B	Yes
11	Biotronik Fortress Introducer	2.280	6	45	6F45B	Yes
12	Biotronik Fortress Introducer	2.550	7	45	7F45B	Yes
13	Merit Prelude Roadster	1.956	5	45	5F45M	Yes
14	Merit Prelude Roadster	2.235	6	45	6F45M	Yes
15	Merit Prelude Roadster	2.565	7	45	7F45M	Yes
16	Cook Flexor Ansel	1.880	5	45	5F45C	Yes
17	Cook Flexor Ansel	2.210	6	45	6F45C	Yes
18	Cook Flexor Ansel	2.540	7	45	7F45C	Yes

### Micro-CT setup and parameters

To investigate the effective inner diameters (eID) at different localizations within the sheaths, an FF35 micro-focus Industrial CT system (Comet Yxlon GmbH, Hamburg, Germany) in an accredited testing laboratory was used. Table 2 shows the chosen scan-parameters, which resulted in a spatial resolution of 4 µm, ensuring optimal contrast of the polymeric inner sheath wall. Due to the different material properties of braided and non-braided sheaths, two different scan protocols have been used. Otherwise, the polymeric part of the braided sheaths would have been invisible when using the scan parameters, which were applied for non-braided sheaths.

**Table 2:** Overview of the Micro-CT measurement parameters for the non-braided and braided sheaths

**Parameters**	**Scan protocol**	
	**Non-braided sheaths**	**Braided sheaths**
Voltage [kV]	43	43
Current [µA]	975	415
Tube	Nanofocus	Microfocus
Binning	2 × 2	2 × 2
Frame rate [Hz]	1.2	2.7
Sensitivity	100	100
Integration time [s]	0.04	0.01
Median filter	5 × 5	5 × 5
Metal artifact reduction	No	Yes

**Fig. 2:**
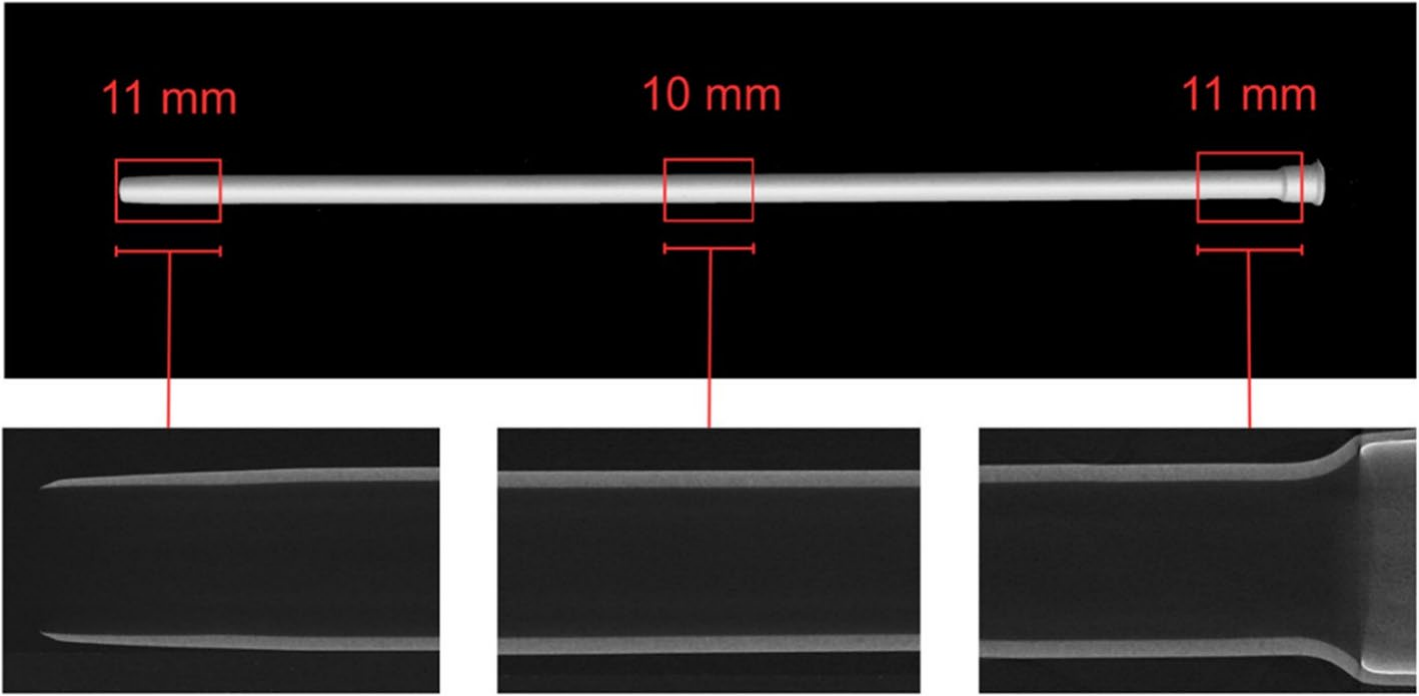
Iso-surface rendering of a complete Micro-CT scan of an exemplary sheath (Terumo Radifocus Introducer II, 7 Fr, 10 cm) with the measurement localizations: aperture/tip, middle, and hub. The proximal and distal locations were measured with an overscan length of 1 mm in addition to the measurement segment of 10 mm

To reduce high-contrast artifacts of the braided sheaths, caused by their metallic components, a metal artifact reduction (MAR) algorithm was applied during reconstruction of the scanned volumes. This algorithm uses threshold-based segmentation to identify metallic regions and suppresses artifacts by using information from neighboring, unaffected projections, thus improving image quality. Furthermore, an edge-preserving 5 × 5 median filter was applied to reduce noise. A graphite tube was used as a sturdy fixture for the measurement of the sheaths, due to its low X-Ray attenuation. Each sheath was positioned free from axial tension in vertical orientation and measured at three specific locations: aperture/tip, middle, and hub. The measurements were acquired with stop-and-go helical scans, that covered a segment of 10 mm at each location (Fig. 2). For the tip/aperture and the hub, 1 mm was added to the scan lengths, to ensure further noise reduction and avoid object cut-offs. The tomogram reconstruction was performed by using an FDK-filtered back-projection algorithm with auto-alignment of the projection images. The reconstruction was executed with CERA (Siemens Healthineers AG, Erlangen, Germany).

### Data analysis

**Fig. 3:**
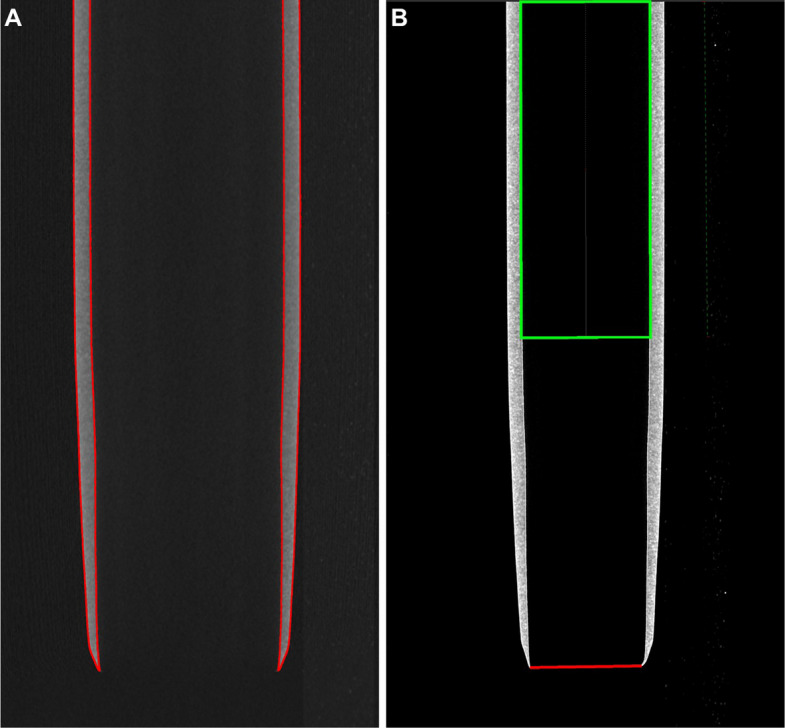
Surface determination of an exemplary sheath (**A**). Based on this, a cylindrical fit (green) over a length of 0.5 cm and a circular fit (red) at the aperture were performed to calculate the inner diameters (**B**). Cylindrical fits were also performed at the middle and the hub region of all sheaths (not shown)

The reconstructed images were analyzed using the *VGStudio* software (version 2022.2, Hexagon AB, Stockholm, Sweden). For the evaluation of the effective inner diameter, surface determination was first performed using a global threshold set at 50% of the value between the polymeric material and the background (Fig. 3A). This ensured recognition of the object and acted as the basis for the following diameter measurements. The effective inner diameter was determined by fitting a cylinder corresponding to the inner surface of the rendered object. This was achieved by distributing detection points on the surface determination of the inner sheath wall. For each region of interest, a regular grid of more than 1000 detection points was automatically and homogeneously distributed on the segmented inner sheath wall. The reported values are given as averages. The sheath’s tips have shown to narrow at the end. Due to this tapered form, a circular fit was chosen to determine the smaller diameter of the aperture (Fig. 3B). For the circular fit at the aperture, 1000 detection points were uniformly sampled along the lumen contour in the corresponding cross-sectional slice. The cylindrical fit at the distal tip was only fitted over a straight segment of 0.5 cm, to avoid erroneous deviations due to improper fitting at the tapered end. It is important to note that the fitting approach was used because it prevents microscopic surface irregularities and image noise from negatively skewing the measurement results.

Additionally, an evaluation of the measurement uncertainty was performed. For the calculation, the cumulative deviation of the distance between the detection points and the fit was determined at 68.27%, which is the determination point of the absolute deviation within the interval of one standard deviation. The measurement uncertainty of Micro-CT coordinate measurements was evaluated in accordance with the GUM framework [10], using the Uncertainty Component Matrix UKM-table software (version 2.10 CT) [11] to establish a comprehensive uncertainty budget. Input quantities included the length measurement deviation obtained from the CT system’s calibration certificate, the number of probing points and repeatability standard deviation per cylinder diameter, the thermal expansion of the measuring device, as well as the workpiece's thermal expansion coefficient and temperature. To determine the thermal expansion coefficient of braided sheaths, which consist of more than one significant material, the Turner model was applied [12]. The Turner model is an analytical model, which determines the coefficient of linear thermal expansion for materials with more than one constituent.

## Results

**Fig. 4:**
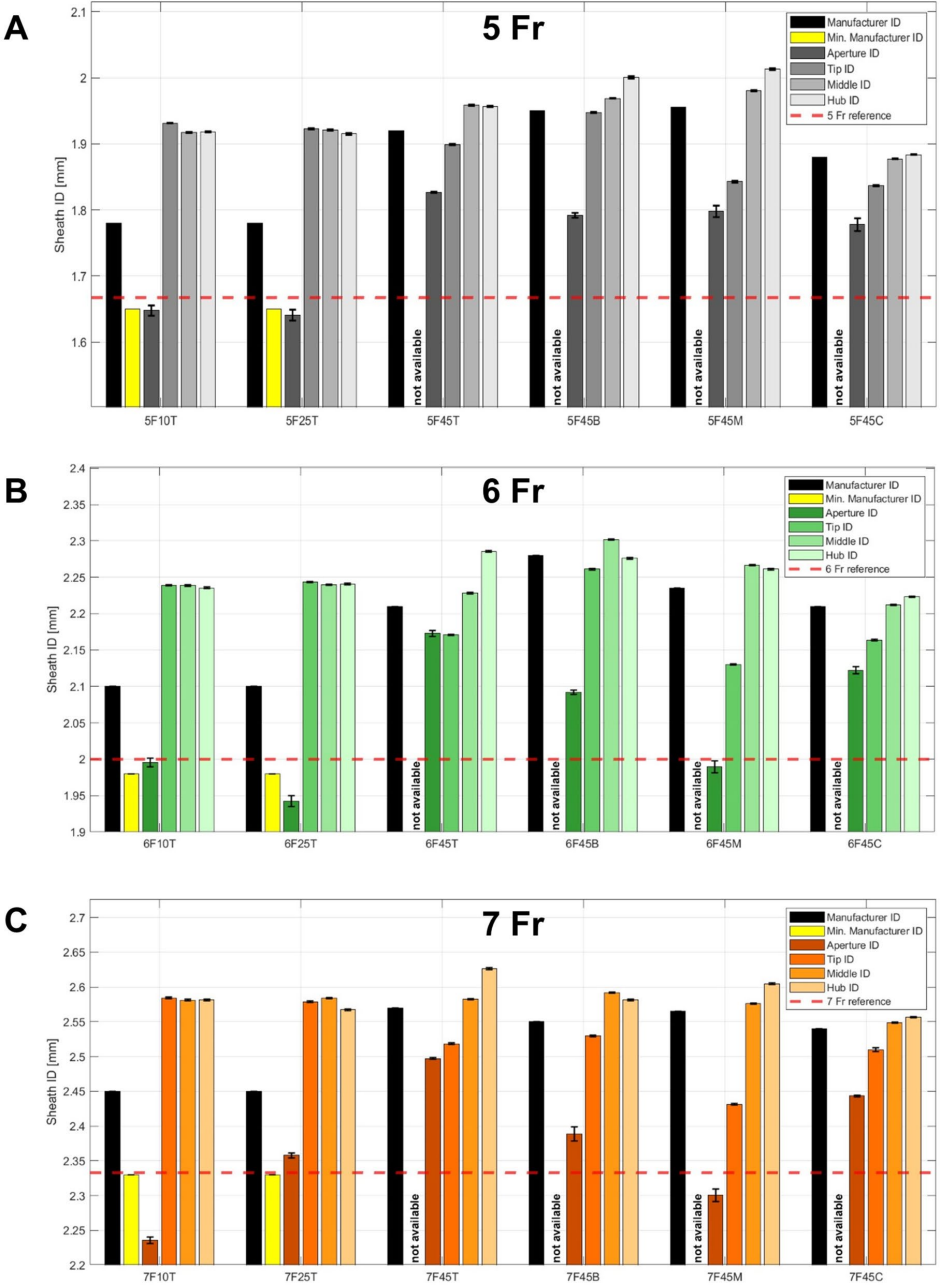
Overview of the absolute measurement results of the **A** 5 Fr, **B** 6 Fr, and **C** 7 Fr sheaths (averages of measured values and standard deviations as error bars). The color scheme represents the established sheath size color code (5 Fr: gray, 6 Fr: green, and 7 Fr: orange). In addition to the measurements at four sheath locations, the manufacturer ID, the minimal manufacturer ID (if available), and the nominal sheath size in Fr are displayed. The nominal inner diameters of the sheaths are illustrated as red dotted lines in all plots. Note that only the relevant segment of the y-axis is shown in the plots

All measured effective sheath IDs (eIDs) showed deviations from the manufacturer-specified IDs (mIDs) (Table 3, Fig. 4).

**Table 3:** Overview of the measured effective inner diameter (ID) deviations (in %) from the manufacturer-specified IDs for all measured sheaths. The first six sheaths are non-braided sheaths, while the twelve sheaths below are braided devices. Measured IDs with negative deviations from the manufacturer specified ID are displayed in bold font

**Abbreviation**	**Measurement location of eID**			
	**Aperture**	**Tip**	**Middle**	**Hub**
5F10T	** − 7.32**	+ 8.50	+ 7.70	+ 7.75
6F10T	** − 4.97**	+ 6.61	+ 6.60	+ 6.45
7F10T	** − 8.74**	+ 5.48	+ 5.35	+ 5.36
5F25T	** − 7.80**	+ 8.01	+ 7.90	+ 7.60
6F25T	** − 7.51**	+ 6.82	+ 6.64	+ 6.70
7F25T	** − 3.76**	+ 5.26	+ 5.47	+ 4.78
5F45T	** − 4.86**	** − 1.10**	+ 1.87	** − 1.90**
6F45T	** − 1.68**	** − 1.78**	+ 0.84	+ 3.43
7F45T	** − 2.84**	** − 2.01**	+ 0.47	+ 2.19
5F45B	** − 8.12**	** − 0.13**	+ 0.95	+ 2.59
6F45B	** − 8.24**	** − 0.83**	+ 0.96	** − 0.17**
7F45B	** − 6.33**	** − 0.80**	+ 1.65	+ 1.23
5F45M	** − 8.07**	** − 5.79**	+ 1.24	+ 2.91
6F45M	** − 10.99**	** − 4.68**	+ 1.41	+ 1.12
7F45M	** − 10.31**	** − 5.21**	+ 0.44	+ 1.54
5F45C	** − 5.44**	** − 2.28**	** − 0.14**	+ 0.20
6F45C	** − 3.96**	** − 2.10**	** − 4.21**	+ 0.60
7F45C	** − 3.81**	** − 1.19**	+ 0.35	+ 0.66

### Measured effective IDs of non-braided sheaths

All non-braided sheaths revealed eIDs at the hub, middle, and tip which were larger than the mIDs. The largest deviation was detected for the 5 Fr/10 cm Terumo sheath with an eID 8.5% larger than the mID. The measured deviations showed an indirect correlation with the sheath diameter, as they were largest for 5 Fr sheaths and smallest for the 7 Fr sheaths. All measurements performed at the sheath tip and aperture revealed eIDs which were smaller than the mIDs. Compared with the minimal IDs (which were only given for Terumo sheaths), the eIDs at the apertures were smaller for the 5 Fr/25 cm, 6 Fr/25 cm, and 7 Fr/10 cm Terumo Radifocus sheaths. In relation to the nIDs in Fr, the eIDs of all Terumo Radifocus sheaths, with exception of the 7 Fr/25 cm Radifocus device, were smaller.

### Measured effective IDs of braided sheaths

The eIDs of the braided sheaths at the hub were larger than the mIDs with two exceptions: 5 Fr/45 cm Terumo Radifocus and 6 Fr/45 cm Biotronik Fortress. All other sheaths were up to 3.4% larger than the mIDs. Most measurements at the sheath middles also revealed eIDs above the mIDs. Only two sheaths from Cook (Flexor Ansel 5 Fr/45 cm and 6 Fr/45 cm) showed negative deviations of up to 4.2%. In contrast, at both the tips and the apertures, all braided sheaths were smaller than the mID. The tested sheaths from Biotronik and Merit showed the largest negative deviations with a maximum of − 10.99% at the aperture of the 6 Fr/45 cm Merit Prelude Roadster sheath. In comparison with the nIDs in Fr, all eIDs of the braided sheaths, except the apertures of 6 Fr/45 cm and 7 Fr/45 cm Merit Roadster, were larger.

## Discussion

In this study, we report systematic discrepancies between manufacturer-reported and measured effective inner diameters of commercially available vascular introducer sheaths. The observed deviations were most pronounced in braided sheaths, particularly at the sheath tip. Our findings provide a plausible explanation for mismatches between nominal sheath size and actual device compatibility in clinical practice and thus offer indications for sufficient size selection.

Despite first approaches of sheathless interventions [13], the usage of sheaths is widely established and crucial for safe and efficient endovascular procedures. The size selection of sheaths is commonly based on nominal size compatibility between sheaths and the anticipated catheters. So far, unexpected resistance during insertion or removal remains a procedural challenge, especially for balloon catheters which are larger after inflation than in the initial crimped state. Thus, the observed negative deviations of the inner diameters of up to 11% seem to be sufficient to increase friction and thus impair device maneuverability. Resulting forceful manipulation can potentially increase the risk of device damage and procedural complications. In addition to clinical routine, inner diameter variations and related increase of friction are potentially important for the precise performance during future robotic-guided interventions [14].

The key finding of our study is the systematic reduction of the measured effective inner diameters compared with manufacturer specifications. This effect was especially pronounced in the apertures of all sheaths and at the tips of braided sheaths. While metallic braiding of polymer sheaths is primarily used to increase mechanical stability, the present data give a hint that this design type may be associated with a reduced usable lumen. Nevertheless, as only non-braided sheaths of a single manufacturer were tested, the results must be interpreted carefully. The systematic nature of these findings suggests a design-related characteristic rather than an unintended deviation. Although the tapered tip design enhances an atraumatic vessel entry, it also represents a very critical region regarding device passage, steerability, and retrieval. Interestingly, a single sheath manufacturer (Terumo) declared a “minimal inner diameter” in addition to the general inner diameter. When acknowledging this parameter, small deviations were only observed for two of the six tested sheaths with an existing minimum manufacturer inner diameter. Consequently, the specification of the diameter reduction, especially caused by the widely established tapered tip design, could relevantly improve the size selection of these sheaths. Particularly when using balloon-mounted stent systems and balloon catheters lacking a low-profile design, it may be advisable in specific cases to choose a sheath size larger than would be expected based on the declared inner sheath diameter.

The observed variability of effective inner diameters along the sheath length highlights that a single manufacturer-reported value does not adequately describe the functional lumen geometry. In addition to the inner diameter, the outer diameter might also be influenced by sheaths with inner diameters larger than specified. Here, the work from Mathis et al. reports variations in the outer diameter across different vendors [8]. Furthermore, an increasing outer sheath diameter was reported to be related to an increasing risk of hematoma and bleeding [4]. In that regard, knowledge of the effective sheath inner diameter is crucial to prevent unnecessary usage of oversized sheaths. A standardized recommendation scheme from the manufacturers would improve this aspect. Furthermore, the device compatibility across different vendors also should be declared in a standardized manner.

Beyond geometric factors, material-related properties influence friction and thereby the functional performance of introducer sheaths, e.g., hydrophilic coatings have been shown to substantially reduce frictional forces of sheaths [15]. However, the extent of contact between sheath and introduced device is primarily determined by the diameter mismatch between the sheath and the inserted catheter, which may outweigh material-related effects.

This work has several limitations which should be discussed. Considering the large number of available sheath types, only a limited number of models, sizes, and manufacturers were evaluated, which may restrict generalizability. As only a single sheath was tested per type, variations across different batches and thus manufacturing consistency are not addressed in our study. Furthermore, in order to ensure metrological traceability, the measurements were performed in an accredited testing laboratory under controlled static, dry, and ambient conditions. As such, the scope of this study does not account for potential thermal or functional variables. In future studies, the measurements should be performed under in vivo-like conditions with 37 °C and liquid environments.

## Conclusion

This study reveals potential clinically relevant deviations between the manufacturer-specified and effective inner diameters of vascular sheaths. These findings, which were especially pronounced at the tips, underscore the importance of considering effective lumen geometry rather than nominal size alone and may explain the need for sheath upsizing in certain procedural settings. Increased transparency and more detailed declaration regarding effective inner diameter could improve sheath selection and procedural safety in endovascular interventions.

## Supplementary Information

Supplementary Material 1: Suppl. Tab. 1. Absolute values of the manufacturer specified inner diameters and the measured effective inner diameters at the aperture, tip, middle and hub for all measured sheaths. The first six sheaths are the non-braided sheaths, whereas the following twelve sheaths below the horizontal line are the braided introducer sheaths.

## Data Availability

The datasets used and/or analyzed during the current study are available from the corresponding author on reasonable request.
