# Supplementary material for: Differences between manufacturer-specified and measured effective inner diameters of vascular introducer sheaths: a micro-CT analysis
Source: CVIR Endovasc. 2026 Jun 6;9:65. doi: 10.1186/s42155-026-00714-7 (PMC13242346; doi:10.1186/s42155-026-00714-7)
Supplement: Supplementary file 1 — Supplementary Material 1: Suppl. Tab. 1. Absolute values of the manufacturer specified inner diameters and the measured effective inner diameters at the aperture, tip, middle and hub for all measured sheaths. The first six sheaths are the non-braided sheaths, whereas the following twelve sheaths below the horizontal line are the braided introducer sheaths. [file 42155_2026_714_MOESM1_ESM.docx]

**Supplementary table**

**Suppl. Tab. 1:** Absolute values of the manufacturer specified inner diameters and the measured effective inner diameters at the aperture, tip, middle and hub for all measured sheaths. The first six sheaths are the non-braided sheaths, whereas the following twelve sheaths below the horizontal line are the braided introducer sheaths**.**

| **Abbreviation** | **Inner Diameter (ID) in mm** | | | | |
| --- | --- | --- | --- | --- | --- |
|  | Manufacturer-specified (mID) | Measured - Aperture | Measured - Tip | Measured - Middle | Measured - Hub |
| **5F10T** | 1.7800 | 1.6497 | 1.9313 | 1.9171 | 1.9180 |
| **6F10T** | 2.1000 | 1.9956 | 2.2388 | 2.2386 | 2.2355 |
| **7F10T** | 2.4500 | 2.2358 | 2.5842 | 2.5811 | 2.5813 |
| **5F25T** | 1.7800 | 1.6412 | 1.9226 | 1.9207 | 1.9153 |
| **6F25T** | 2.1000 | 1.9422 | 2.2432 | 2.2395 | 2.2406 |
| **7F25T** | 2.4500 | 2.3578 | 2.5788 | 2.5839 | 2.5672 |
| **5F45T** | 1.9200 | 1.8266 | 1.8988 | 1.9583 | 1.9564 |
| **6F45T** | 2.2100 | 2.1728 | 2.1707 | 2.2285 | 2.2857 |
| **7F45T** | 2.5700 | 2.4971 | 2.5183 | 2.5822 | 2.6263 |
| **5F45B** | 1.9500 | 1.7916 | 1.9474 | 1.9686 | 2.0006 |
| **6F45B** | 2.2800 | 2.0922 | 2.2610 | 2.3018 | 2.2761 |
| **7F45B** | 2.5500 | 2.3887 | 2.5296 | 2.5921 | 2.5813 |
| **5F45M** | 1.9560 | 1.7982 | 1.8428 | 1.9802 | 2.0130 |
| **6F45M** | 2.2350 | 1.9894 | 2.1303 | 2.2665 | 2.2610 |
| **7F45M** | 2.5650 | 2.3005 | 2.4313 | 2.5764 | 2.6046 |
| **5F45C** | 1.8800 | 1.7778 | 1.8371 | 1.8774 | 1.8838 |
| **6F45C** | 2.2100 | 2.1224 | 2.1636 | 2.2117 | 2.2233 |
| **7F45C** | 2.5400 | 2.4433 | 2.5098 | 2.5490 | 2.5567 |
